# Automatic Recommender System of Development Platforms for Smart Contract–Based Health Care Insurance Fraud Detection Solutions: Taxonomy and Performance Evaluation

**DOI:** 10.2196/50730

**Published:** 2024-10-18

**Authors:** Rima Kaafarani, Leila Ismail, Oussama Zahwe

**Affiliations:** 1 Intelligent Computing and Communication Systems Laboratory, Computer Science Department, American University of Culture and Education Beirut Lebanon; 2 Intelligent Distributed Computing and Systems Laboratory, Department of Computer Science and Software Engineering, College of Information Technology, United Arab Emirates University Al Ain, Abu Dhabi United Arab Emirates; 3 Vectrawave Device Lannion France

**Keywords:** blockchain, blockchain development platform, eHealth, fraud detection, fraud scenarios, health care, health care insurance, health insurance, machine learning, medical informatics, recommender system, smart contract, taxonomy

## Abstract

**Background:**

Health care insurance fraud is on the rise in many ways, such as falsifying information and hiding third-party liability. This can result in significant losses for the medical health insurance industry. Consequently, fraud detection is crucial. Currently, companies employ auditors who manually evaluate records and pinpoint fraud. However, an automated and effective method is needed to detect fraud with the continually increasing number of patients seeking health insurance. Blockchain is an emerging technology and is constantly evolving to meet business needs. With its characteristics of immutability, transparency, traceability, and smart contracts, it demonstrates its potential in the health care domain. In particular, self-executable smart contracts are essential to reduce the costs associated with traditional paradigms, which are mostly manual, while preserving privacy and building trust among health care stakeholders, including the patient and the health insurance networks. However, with the proliferation of blockchain development platform options, selecting the right one for health care insurance can be difficult. This study addressed this void and developed an automated decision map recommender system to select the most effective blockchain platform for insurance fraud detection.

**Objective:**

This study aims to develop smart contracts for detecting health care insurance fraud efficiently. Therefore, we provided a taxonomy of fraud scenarios and implemented their detection using a blockchain platform that was suitable for health care insurance fraud detection. To automatically and efficiently select the best platform, we proposed and implemented a decision map–based recommender system. For developing the decision-map, we proposed a taxonomy of 102 blockchain platforms.

**Methods:**

We developed smart contracts for 12 fraud scenarios that we identified in the literature. We used the top 2 blockchain platforms selected by our proposed decision-making map–based recommender system, which is tailored for health care insurance fraud. The map used our taxonomy of 102 blockchain platforms classified according to their application domains.

**Results:**

The recommender system demonstrated that Hyperledger Fabric was the best blockchain platform for identifying health care insurance fraud. We validated our recommender system by comparing the performance of the top 2 platforms selected by our system. The blockchain platform taxonomy that we created revealed that 59 blockchain platforms are suitable for all application domains, 25 are suitable for financial services, and 18 are suitable for various application domains. We implemented fraud detection based on smart contracts.

**Conclusions:**

Our decision map recommender system, which was based on our proposed taxonomy of 102 platforms, automatically selected the top 2 platforms, which were Hyperledger Fabric and Neo, for the implementation of health care insurance fraud detection. Our performance evaluation of the 2 platforms indicated that Fabric surpassed Neo in all performance metrics, as depicted by our recommender system. We provided an implementation of fraud detection based on smart contracts.

## Introduction

### Rationale

Health care insurance fraud presents a significant challenge for both the medical industry and government bodies. It represents a serious concern for the insurance industry due to fraud’s financial impact on policyholders and insurance companies. According to the National Health Care Anti-Fraud Association, health care insurance fraud leads to the loss of tens of billions of dollars annually [1]. In 2020, according to the US Department of Justice, a noteworthy accomplishment in combating health care insurance fraud, recovering US $2.7 billion through settlements and judgment, was announced; however, it represented a significant 50% increase compared to the previous year [2]. Furthermore, the global health care insurance fraud analytics market demonstrates substantial growth, rising from US $2.43 billion in 2022 to US $3.09 billion in 2023, reflecting a compound annual growth rate of 27% [3]. On the other hand, health insurance is crucial to ensure people’s lives due to the high cost of medical treatments. The advantages of health insurance are being threatened by theft and fraudulent claims. With the increasing number of patient demands for health insurance, manual auditing for validating and pinpointing fraud is no longer efficient. Therefore, it is essential to create an automatic and efficient system that detects fraud.

Hence, machine learning solutions for detecting fraud that rely on data sets to train models for fraud detection have been introduced [4,5]. However, they raise ethical concerns as the trained models could be biased toward the majority [6], and there are also privacy and security issues [7] due to the potential compromise of sensitive personally identifiable information of patients. These considerations would have severe consequences, including reputational damage to insurance firms. Machine learning models should rely on high-quality data [8]. Therefore, they are not trustworthy so far. Recently, blockchain has emerged as a decentralized technology to implement secure transactions in a peer-to-peer network. It consists of a series of interconnected blocks of transactions. Each block contains data and is secured through cryptographic measures, such as hash functions and asymmetric encryption [9]. Transactions occur between nodes in a peer-to-peer network without the need for a central authority. All transactions are recorded in an immutable ledger, and peers can only add to the ledger, not alter or delete any previously recorded information [10]. When a new node joins the network, it downloads a copy of the ledger. Before adding a block to the blockchain, a consensus is reached among peers. In addition, blockchain can execute smart contracts [11].

Blockchain has demonstrated its potential in various domains, including the health care system [12,13]. In particular, smart contracts in a blockchain were introduced as self-executing agents based on the transactions being executed [14]. However, there is a proliferation of blockchain development platforms in the literature with various characteristics, imposing challenges for software developers to determine suitable platforms that include the functionalities needed to implement insurance fraud detection solutions based on smart contracts. In this paper, we propose an automated decision map recommender system specifically designed to select the most suitable blockchain platform among the proposed platforms in the literature. We exemplify the use of our proposed recommender system by implementing smart contract–based solutions for insurance fraud detection on the selected platform. The main contributions of this research are as follows:

- We proposed and developed an innovative, adaptive, and automated recommender system based on our proposed decision map. The map evaluates blockchain platforms, considering selected and categorized blockchain features. to suggest the most suitable platform. The system is flexible and responsive to changes, ensuring that, if a platform becomes unavailable or gains new features, it will generate updated results accordingly.
- We introduced a decision-making map recommender system that allows us to identify the best blockchain platform that is adequate for the implementation of health care insurance fraud detection. The decision map is generic and can be applied to any other domain.
- We developed a taxonomy of blockchain development platforms, used to determine the characteristics of the platforms that are available for implementing applications in the health insurance field. The platform taxonomy is based on the investigation of 102 blockchain platforms and their applications domains in the literature.
- We exemplified the applicability of our automated decision map recommender system by developing and implementing blockchain smart contracts for the detection of 12 fraud scenarios.
- We evaluated the implementation of our recommender system by applying it to 42 blockchain platforms. Consequently, we developed and implemented the detection of fraud on the top 2 platforms recommended by our decision-making map recommender system and evaluated their performances.
- We made the recommender system toolkit and source code available on GitHub for blockchain developers.

### Related Works

To our knowledge, no work in the literature has automated the selection of a suitable blockchain development platform for a specific use case, such as health insurance fraud detection. In the study by Farshidi et al [15], the authors divided blockchain features into four categories: (1) *must-have*, which indicates that the platform needed to include the specified blockchain feature to be deemed suitable; (2) *should-have*, which implies that the defined blockchain features are highly recommended; (3) *could-have*, which represent optional blockchain features; and (4) *won’t-have*, which meant to list the features that are not required by the developer. However, this method may not be precise as blockchain platforms often possess multiple features; for instance, some platforms offer various consensus mechanisms. Thus, classifying a single consensus into the *won’t-have* category could unjustly disqualify a blockchain platform that might otherwise be suitable for the use case. In addition, our system implements software that can be used by any clinic or hospital interested in adopting blockchain platforms. Moreover, the system is adaptive as it allows for adding both blockchain platforms and features as well as the modification of existing ones.

Some works have introduced machine learning and deep learning models for identifying fraud and overcoming the constraints of manual detection methods. Learning models automate the detection process and enhance the analysis of patterns. As shown in Table 1, the study by Lu et al [16] proposes a deep learning graph model, which relies on an attributed heterogeneous information network with a hierarchical attention mechanism. The study by Sowah et al [17] develops a decision support system using Genetic Support Vector Machines to enhance the detection and classification of health insurance fraud in Ghana. The study by Settipalli et al [18] proposes an unsupervised multivariate analysis model named Weighted MultiTree Density-Based Clustering. However, the use of artificial intelligence for detecting health care insurance fraud has raised security concerns, largely due to the sensitive client data used in training the models, consequently suffering from privacy and security issues. In addition, these works do not consider the bias introduced by the use of machine learning or deep learning algorithms. As a result, our emphasis will be on solutions that leverage smart contracts, which are self-executing agreements with predefined rules that activate when conditions are fulfilled. These contracts are immutable, meaning that they cannot be altered once deployed, providing a secure and privacy-preserving blockchain solution for detecting health care insurance fraud. Moreover, throughput, latency, and Central Processing Unit (CPU) and memory use have not been taken into account in the aforementioned works.

Therefore, researchers and developers are turning toward privacy-preserving and secure blockchain-based solutions that incorporate smart contracts for the detection of health insurance fraud. These contracts execute automatically under set conditions once deployed on the blockchain, benefiting from the platform’s immutability, decentralization, and transparency, and cannot be changed after they are set up. The study by Mackey et al [19] focuses on determining whether a claim adheres to the applicable provisions of the health care insurance policy. The study by Saldamli et al [20] proposes a solution for preventing health insurance fraud by using 2 fraud scenarios. The study by Liu et al [21] uses the Ethereum blockchain to develop a framework for recording claim data and transaction patients as validators to assist in the detection of fraud. However, none of these works takes into account all possible fraud scenarios; the quality of service of fraud detection in terms of throughput and latency; or computing resource use, such as CPU and memory. In addition, the use of blockchain platforms is unjustified, and the choice of the development platform is not justified. Our recommender system is adaptive to the evolution of blockchain platforms, offering a comprehensive approach. Furthermore, smart contracts are portable and can operate across different platforms. In this study, we implemented smart contracts based on a blockchain development platform that is selected by our adaptive automatic decision map recommender system. On the basis of these fraud scenarios, we implemented smart contracts for insurance fraud detection on the top 2 blockchain development platforms selected by our recommender system. The strengths and weaknesses of recent works using blockchain development platforms for detecting health care insurance fraud are summarized in Table 2**.**

**Table 1:** Summary of related works on fraud detection machine learning (ML) and deep learning (DL) algorithms in health insurance claims.

Algorithm under study	Number of fraud scenarios detected	Considering privacy and security	Considering bias issue	ML or DL	Throughput	Latency	CPU^a^ use	Memory use	Data set	Metrics
MHAMFD^b^ [16]	NR^c^	X^d^	X	DL	X	X	X	X	Medical-1: balanced data set with a ratio of positive to negative samples of 1:2 Medical-2: unbalanced data set with a ratio of positive to negative samples of approximately 1:70	Medical-1: Accuracy: 0.8961 F1-score: 0.8694 Medical-2: F1-score: 0.8361 Recall: 0.8764 Precision: 0.9194
GSVMs^e^ [17]	NR	X	X	ML	X	X	X	X	100-claim data set 300-claim data set 500-claim data set 750-claim data set 1000-claim data set	100-claim data set accuracy: 71.43% 300-claim data set accuracy: 95.45% 500-claim data set accuracy: 99.18% 750-claim data set accuracy: 82.56% 1000-claim data set accuracy: 90.91%
WMTDBC^f^ [18]	NR	X	X	ML	X	X	X	X	The data set used in the study was the claims data submitted by health care providers under the US Medicare CMS^g^ Part B health care program	Overall accuracy ranged from 0.857 to 0.946.

^a^CPU: Central Processing Unit.

^b^MHAMFD: Multilevel Hierarchical Attention Mechanism for Fraud Detection.

^c^NR: not reported.

^d^X: not considered.

^e^GSVM: Genetic Support Vector Machine.

^f^WMTDBC: Weighted MultiTree Density-Based Clustering.

^g^CMS: Centers for Medicare & Medicaid Services.

**Table 2:** Summary of related works on blockchain-based health care insurance fraud detection.

Study	Throughput	Latency	CPU^a^ use	Memory use	Fraud scenarios considered, N	Smart contract	Recommender system	Platform	Reason for choosing the platform
Mackey et al [19]	X^b^	X	X	X	1	✓^c^	X	Ethereum	NR^d^
Saldamli et al [20]	X	X	X	X	2	✓	X	BigchainDB	NR
Liu et al [21]	X	X	X	X	3	✓	X	NR	NR
Our work	✓	✓	✓	✓	12	✓	✓	Hyperledger Fabric and Neo	On the basis of our proposed decision-making map recommender system tailored to health care insurance fraud detection

^a^CPU: Central Processing Unit.

^b^X: not considered.

^c^✓: considered.

^d^NR: not reported.

## Methods

### Overview

**Figure 1:**
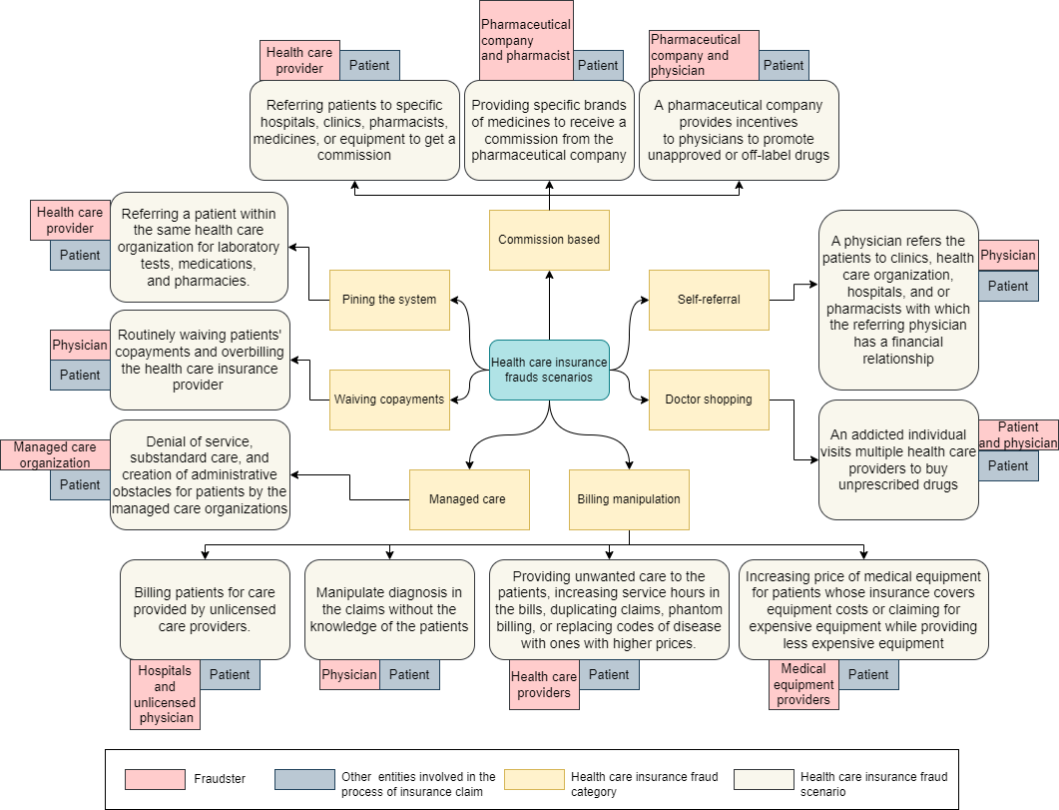
Taxonomy of health care insurance fraud scenarios [22].

The taxonomy of blockchain platforms was based on reviewing published research articles and white papers that mentioned blockchain platforms. Our study revealed 102 blockchain platforms that we classified according to the application domains they were developed for, such as financial services, social media, Internet of Things, and platforms that can be used across several domains. In addition, we gathered information on various features, such as whether the platform is open source, the consensus mechanism used, the type of blockchain used, and the availability of smart contracts. For the detection of health care insurance fraud, the fraud scenarios were based on the study by Ismail and Zeadally [22], which proposes a taxonomy of 12 fraud scenarios that are divided into 7 categories, as shown in Figure 1. The first category is *commission-based*, which includes 3 fraud scenarios. The first scenario involves a health care provider directing patients to specific hospitals, clinics, pharmacies, medications, or equipment suppliers in return for a commission. The second fraud scenario involves pharmacies dispensing specific brands of medicines in exchange for commissions from pharmaceutical companies. The third fraud scenario involves pharmaceutical companies offering incentives to physicians to recommend unapproved or off-label drugs. The second category is *Pinning the System*, which involves health care providers guiding patients to internal entities such as laboratories or pharmacies to keep profits within the organization. The third category, *Waiving Copayments*, is where the physician regularly waives patients’ copayments and overcharges the health care provider. The fourth category, *Managed Care*, consists of organizations limiting costs by denying necessary care, providing substandard treatment, and creating administrative barriers for patients. The fifth category, *Billing Manipulation*, consists of 4 fraud scenarios. The first involves unlicensed hospitals and physicians billing patients for care. The second scenario occurs when a physician alters a diagnosis on a claim without the patient’s knowledge. The third scenario involves health care providers offering unnecessary care, inflating service hours, submitting duplicate claims, phantom billing, or substituting diagnosis codes for higher reimbursements. The final scenario involves medical equipment providers inflating prices for insured patients or claiming expensive equipment while supplying cheaper alternatives. The sixth category, *Physician Shopping*, involves patients consulting multiple health care providers to obtain prescriptions for nonmedical use. Finally, the seventh category, *Self-referral*, occurs when physicians direct patients to clinics or health care facilities in which they have a financial interest, potentially leading to conflicts of interest.

### Ethical Considerations

The study does not involve any personal or patient-related data, focusing solely on blockchain platforms features. As no human subjects are included and no identifiable information is used, the research does not require ethics review board assessment, in accordance with institutional and regional guidelines for nonhuman subjects research.

## Results

### Decision-Making Map Recommender System for Selecting the Best Blockchain Platform for Health Care Insurance Fraud Detection

#### Overview

The proliferation of blockchain platforms has led to a multitude of choices for developers. However, it is important to note that the various blockchain platforms available today have different features, capabilities, and use cases [23,24]. Therefore, developers need to evaluate the available options and select the platform that best fits their specific needs. In this section, we provide an overview of our taxonomy, which encompasses 102 blockchain platforms. Subsequently, we present our feature-based decision map recommender system to select the best platform.

#### Taxonomy of Blockchain Platforms

**Figure 2:**
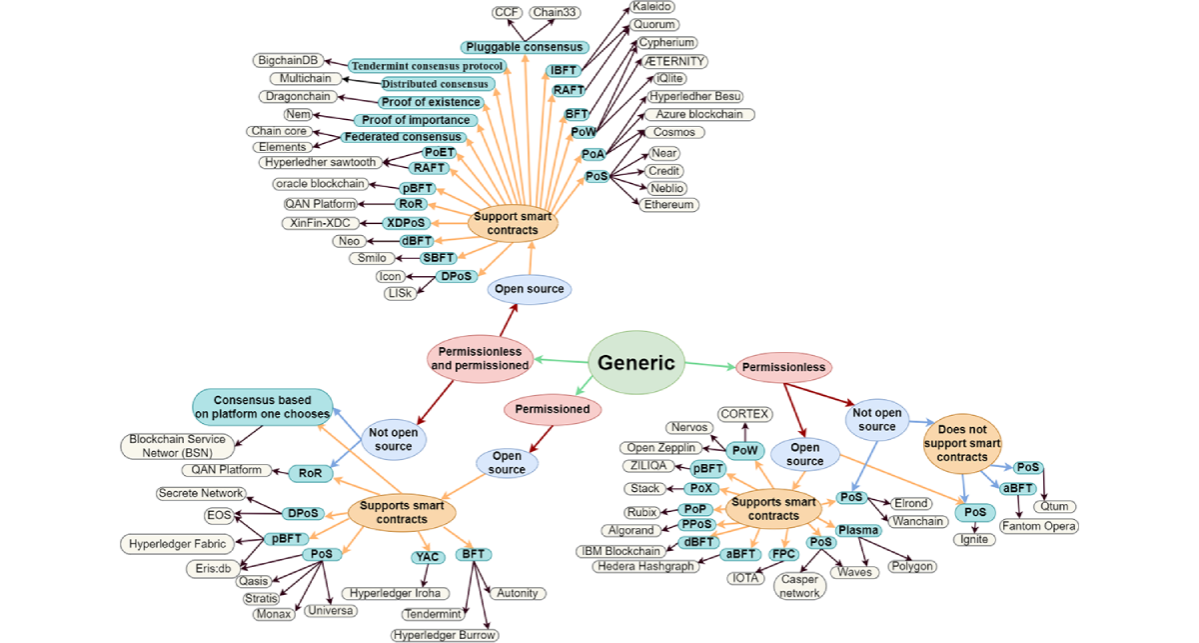
Graphical representation of the taxonomy of blockchain platforms—generic blockchain platforms. aBFT: Asynchronous Byzantine Fault Tolerant; BFT: Byzantine Fault Tolerance; dBFT: Delegated Byzantine Fault Tolerance; DPoS: Delegated Proof of Stake; FPC: Fast Probabilistic Consensus; IBFT: Istanbul Byzantine Fault Tolerance; pBFT: Practical Byzantine Fault Tolerance; PoA: Proof of Authority; PoET: Proof of Elapsed Time; PoP: Proof of Pledge; PoS: Proof of Stake; PoW: Proof of Work; PoX: Proof of Transfer; PPoS: Pure Proof of Stake; RAFT: Reliable, Replicated, Redundant, and Fault-Tolerant; RoR: Return on Reputation; SBFT: Smilo Byzantine Fault Tolerance; XDPoS: Extended Delegated Proof of Stake; YAC: Yet Another Consensus.

In 2008, Bitcoin [25] made its debut, and the subsequent addition of smart contract technology by Ethereum [26] contributed significantly to the rapid growth and development of blockchain technology. As a result, >100 distinct blockchain platforms were developed for various purposes. To provide a comprehensive understanding of these platforms, we present a taxonomy of 102 blockchain platforms, which we organized based on their respective application domains. Along with the application domain, our classification takes into account the open-source nature of the platform, the consensus mechanism used, the type of blockchain, and the platform’s capability to support smart contract development. The taxonomy of blockchain platforms is presented as a graph. Figure 2 shows an overview of the different generic blockchain platforms that can be used to build a wide range of applications.

**Figure 3:**
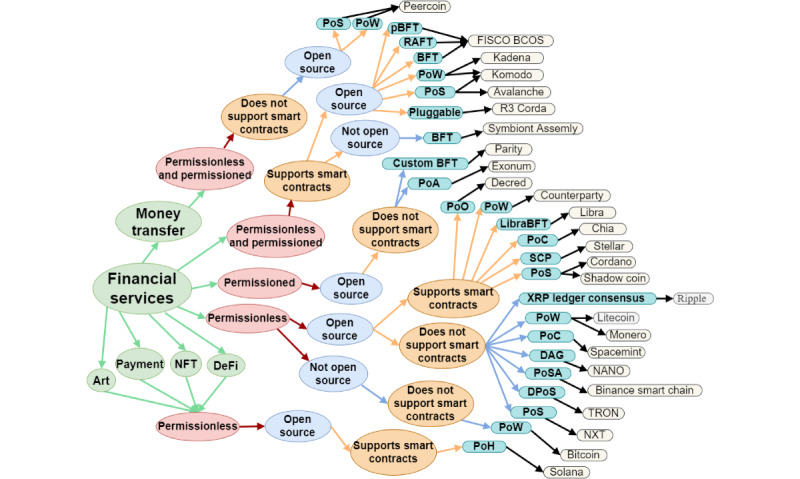
Graphical representation of the taxonomy of blockchain platforms—blockchain platforms dedicated to financial services. aBFT: Asynchronous Byzantine Fault Tolerant; BFT: Byzantine Fault Tolerance; DAG: Directed Acyclic Graph; dBFT: Delegated Byzantine Fault Tolerance; DeFi: Decentralized Finance; DPoS: Delegated Proof of Stake; FPC: Fast Probabilistic Consensus; IBFT: Istanbul Byzantine Fault Tolerance; LibraBFT: Libra Byzantine Fault Tolerance; NFT: None Fungible Tokens; pBFT: Practical Byzantine Fault Tolerance; PoA: Proof of Authority; PoC: Proof of Capacity; PoET: Proof of Elapsed Time; PoH: Proof of History; PoO: Proof of Ownership; PoP: Proof of Pledge; PoS: Proof of Stake; PoSA: Proof of Staked Authority; PoW: Proof of Work; PoX: Proof of Transfer; PPoS: Pure Proof of Stake; RAFT: Reliable, Replicated, Redundant, and Fault-Tolerant; SBFT: Smilo Byzantine Fault Tolerance; SCP: Stellar Consensus Protocol; XDPoS: Extended Delegated Proof of Stake; XRP: Ripple; YAC: Yet Another Consensus.

**Figure 4:**
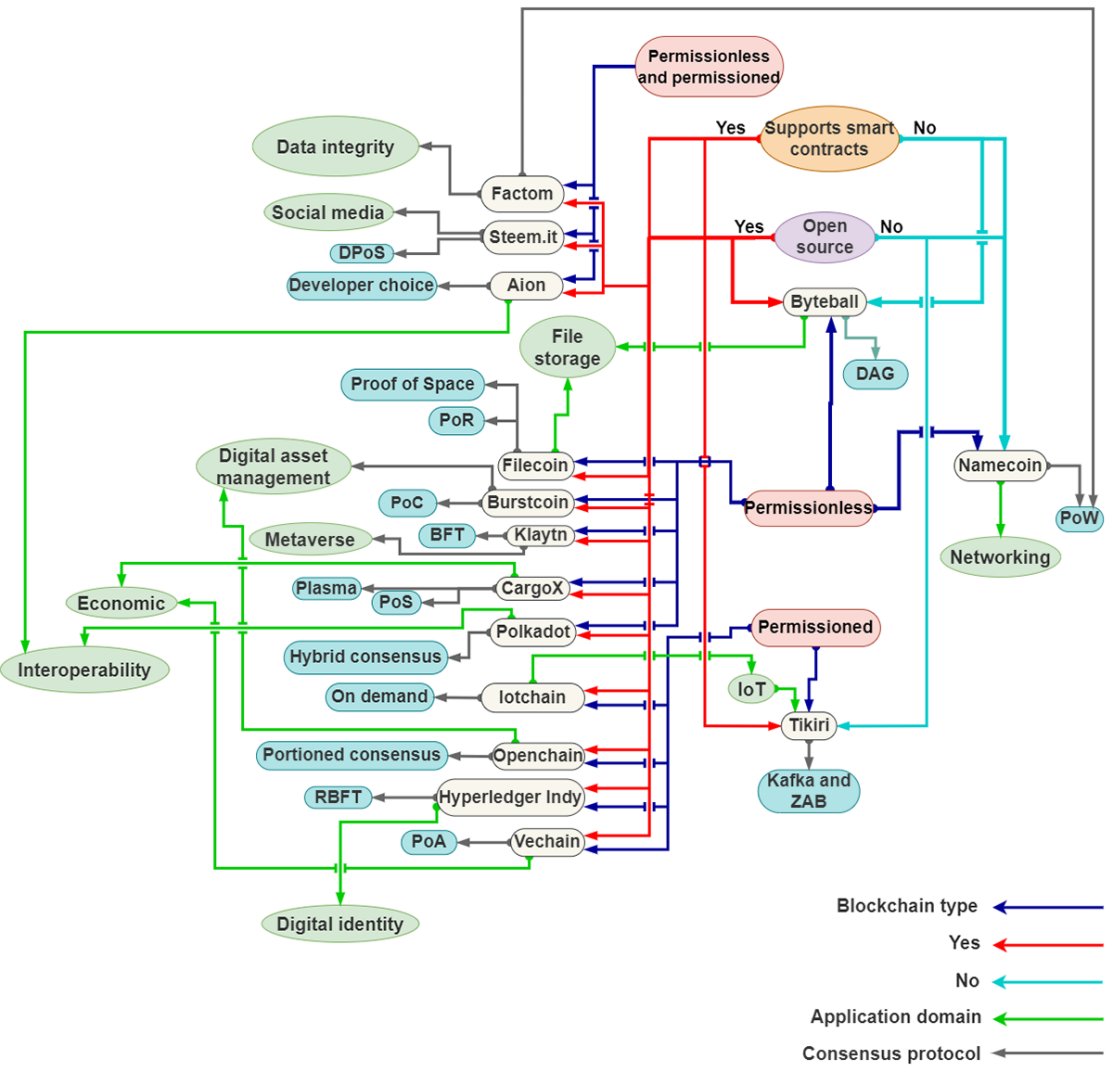
Graphical representation of the taxonomy of blockchain platforms—blockchain platforms mapped to specific application domains. BFT: Byzantine Fault Tolerance; DAG: Directed Acyclic Graph; DPoS: Delegated Proof of Stake; IoT: Internet of Things; PoA: Proof of Authority; PoC: Proof of Capacity; PoR: Proof of Randomness; PoS: Proof of Stake; PoW: Proof of Work; RBFT: Redundant Byzantine Fault Tolerance; ZAB: ZooKeeper Atomic Broadcas.

Figure 3 presents the blockchain platforms that have been specifically designed for financial services, whereas Figure 4 presents the platforms that are tailored to meet the needs of a particular application domain. These platforms offer specialized features and functionality to cater to the specific needs of their respective industries or sectors, thus providing a more specialized and customized solution for these specific use cases.

#### Decision-Making Map–Based Recommender System

**Figure 5:**
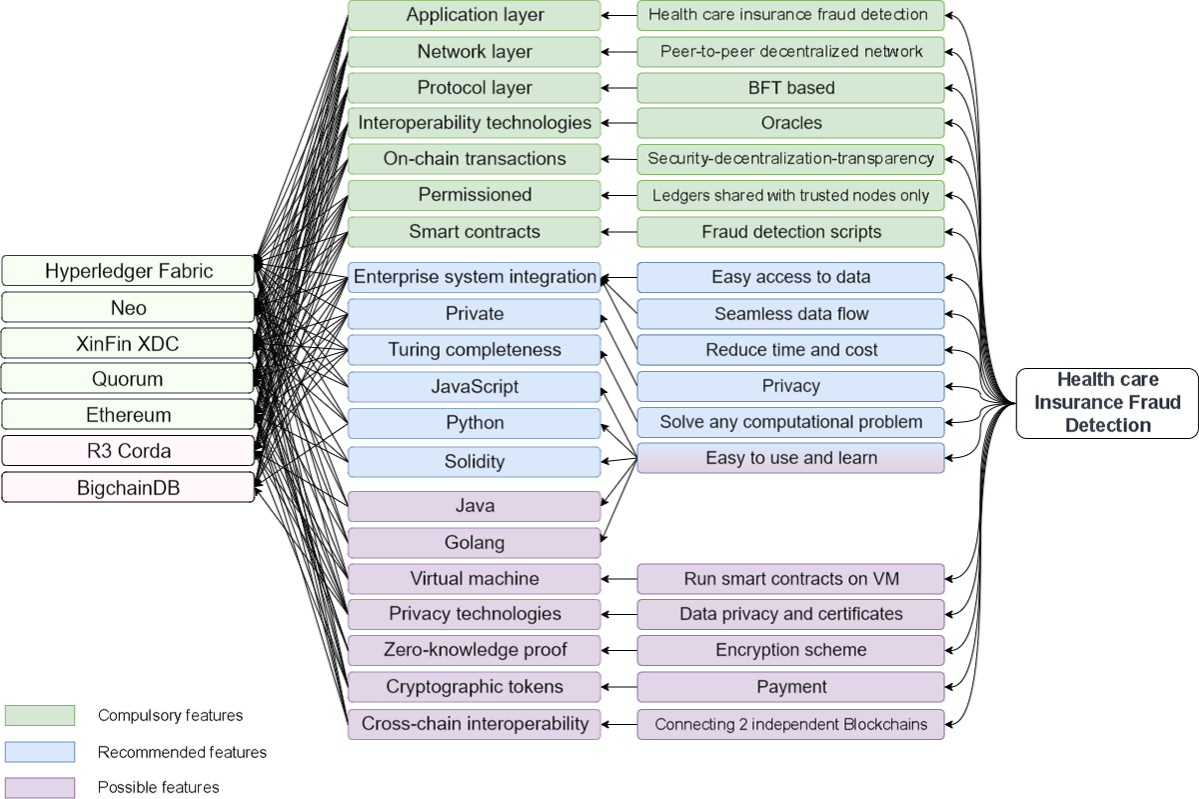
Proposed decision-making map for selecting a platform for health care insurance fraud detection. BFT: Byzantine Fault Tolerance; VM: Virtual Machine.

While a blockchain platform selection method was proposed in the study by Farshidi et al [15], it included unnecessary categories of features and did not specifically focus on the detection of insurance fraud. Therefore, we propose a decision-making map that is tailored specifically to health care insurance fraud detection solutions. It classifies blockchain features into 3 main categories: *compulsory features*, which are essential to the platform; *mandatory features*, which are sufficient; and *possible features*, which are desirable but not necessary. These categories differ in weight, which determines the value of one feature over another. As shown in Figure 5, our map offers a targeted approach to selecting a blockchain platform for developing health insurance fraud detection mechanisms.

In the health care insurance domain, privacy is a crucial aspect as insurance companies deal with sensitive patient data [12]. Several research works in health care have implemented blockchain technology to ensure integrity, accountability, and nonrepudiation in the claim process [19,22]. The study by Ismail and Zeadally [22] proposes a blockchain system for health care insurance antifraud that ensures trusted medical process information entry and reading as well as a data privacy protection scheme. In the study by Mackey et al [19], a blockchain system is proposed and implemented to prevent counterfeiting in health care insurance, providing a secure and private system.

To implement health care insurance fraud detection using blockchain, we should select features that ensure privacy, such as on-chain transactions and permissioned platforms. This is in addition to other technical features that should be available in the platform, such as the smart contract and user interface development tool features. In summary, we determined the most suitable features in terms of both their relevance to the task of health care insurance fraud detection and the technical capabilities of the platforms. We divided these features into 3 categories, which are compulsory, mandatory, and possible features (Textbox 1).

On the basis of the aforementioned selected features (compulsory, mandatory, and possible), our enforced decision-making map selects 42 platforms out of 102. This extraction of 42 platforms is derived from our proposed taxonomy of blockchain platforms. This taxonomy maps the blockchain platforms into their corresponding application domains and blockchain features. To ensure the privacy and security of patient files, the decision map recommender system selects the blockchain platforms that meet these specific criteria. Therefore, the selection process excludes platforms that are based on permissionless blockchain type, which is open to the public and may compromise data confidentiality. Instead, the recommender system prioritizes platforms that are suited for generic application domains and financial services, as per our taxonomy. In addition, the recommender system focuses on platforms that support the development of smart contracts.

After identifying the relevant blockchain features for health care insurance fraud detection, the recommender system initiates a mapping process to match each feature with the platforms that support it. Figure 5 illustrates the outcomes of this mapping. Initially, after organizing the blockchain features into categories, the recommender system proceeds to map each feature with its corresponding functionality. Next, the recommender system maps the features to the blockchain platforms. On the basis of this, it determines the suitability of each platform. Only the platforms that have all the compulsory features are considered suitable. As shown in Figure 5, platforms R3 Corda and BigchainDB were eliminated from consideration due to their lack of some of the compulsory features. Our mapping process revealed that Hyperledger Fabric [27] was the most optimal platform, followed by Neo [28], XinFin XDC [29], Quorum [30], and Ethereum. These results demonstrate the effectiveness of our mapping process in identifying the ideal blockchain platform for this specific use case.

Table 3 streamlines the mapping process for health care insurance fraud detection by listing the top 5 platforms and highlighting the selected features. The table is designed to simplify the decision-making map by providing a concise and easy-to-read format for comparing the features.

**Definition of compulsory, mandatory, and possible features.**

**Compulsory features**

- Application layer: this capability enables the creation of a user interface and the execution of smart contracts for health care insurance.
- Network layer: enables the establishment of a peer-to-peer decentralized network.
- Protocol layer: enables the selection of a consensus protocol. We used Byzantine-based consensus protocols because they prevent the case of a failing or malicious node [11].
- Interoperability technologies: technologies such as Oracle that facilitate the integration of data from off-chain resources into smart contracts.
- On-chain transaction: the transaction is conducted on the main blockchain for increased security, decentralization, and transparency.
- Permissioned blockchain: this type of blockchain limits access to the ledger to a select group of trusted nodes.
- Smart contracts: enables the development of algorithms that can identify health care insurance fraud.

**Mandatory features**

- Enterprise system interrogation: provides easy access to data, seamless data flow, and time and cost savings.
- Private: this type of blockchain network is only accessible to authenticated users.
- Turing completeness: the virtual machine of the blockchain platform is capable of solving any computational problem.
- JavaScript, Python, and Solidity: these languages are specifically mentioned because they are intuitive and easily learned by programmers.

**Possible features**

- Java and Golang: these languages, similar to the 3 mentioned in the mandatory features list, are intuitive and easily learned by programmers.
- Virtual machine: it is used to execute smart contracts.
- Privacy technology: ensures data privacy and certifies the eligibility of peers to participate in the network, particularly when handling sensitive patient data.
- Zero-knowledge proof: this encryption scheme allows one party (the prover) to assure another party (the verifier) that they know a certain value (X) without revealing the value itself.
- Cryptographic token: these tokens have the potential to be used as a means of payment.
- Cross-chain interoperability: this feature enables the connection of 2 separate blockchains to facilitate information exchange.

**Table 3:** Decision-making map results simplified.

Category and feature name			Hyperledger Fabric		Neo		Ethereum		Quorum		XinFin XDC
**Compulsory features**											
	Application layer	✓		✓		✓		✓		✓	
	Interoperability technology	✓		✓		✓		✓		✓	
	Network layer	✓		✓		✓		✓		✓	
	On-chain transaction	✓		✓		✓		✓		✓	
	Permissioned blockchain	✓		✓		✓		✓		✓	
	Protocol layer	✓		✓		✓		✓		✓	
	Smart contract	✓		✓		✓		✓		✓	
**Mandatory features**											
	Enterprise system integration	✓		✓		✓		✓		✓	
	JavaScript	✓		✓						✓	
	Private	✓		✓		✓		✓		✓	
	Python	✓		✓						✓	
	Solidity	✓				✓		✓			
	Turing completeness	✓		✓		✓		✓		✓	
**Possible features**											
	Zero-knowledge proof	✓				✓		✓			
	Virtual machine	✓		✓		✓		✓		✓	
	Java	✓		✓							
	Golang	✓		✓				✓			
	Cryptographic token	✓		✓		✓				✓	
	Cross-chain interoperability	✓		✓		✓				✓	
	Privacy technology	✓		✓		✓		✓		✓	

#### Use Case Diagram for the Recommender System

**Figure 6:**
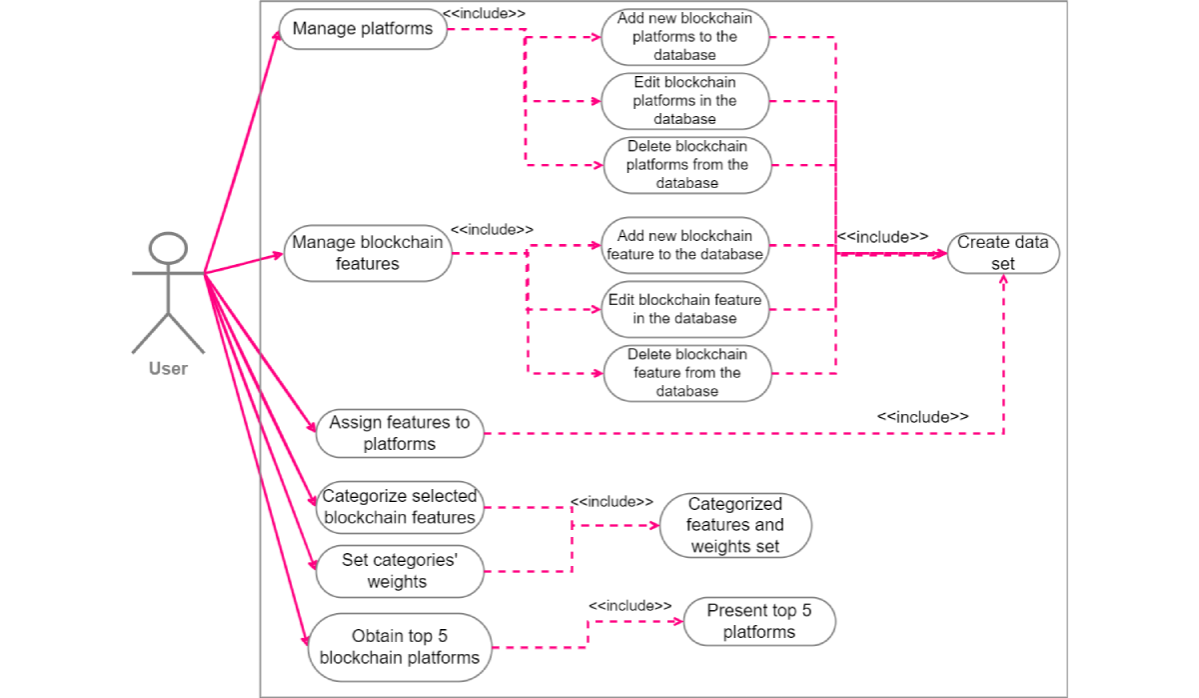
Recommender system use case diagram.

Figure 6 illustrates the use case diagram of our recommender system. Users can perform actions such as adding, editing, and deleting blockchain platforms and features. Following that, they are required to select their desired features, categorize them, and assign weights to mandatory and possible features. Ultimately, users will receive the outcome of the most suitable blockchain platform for their specific use case based on the chosen features.

#### Decision-Making Map Recommender System Implementation

In this subsection, we present our implementation of the decision map recommender system, which is a desktop software solution that provides a streamlined and efficient method to select the most suitable blockchain platform for a specific use case. Our software uses WinForms C# technology (.NET Foundation) and SQL as the database to deliver a user-friendly experience and recommend the top blockchain platforms. To demonstrate the effectiveness of our software, we used it to identify the top 5 blockchain platforms that are most suitable for health care insurance fraud detection.

Textbox 2 defines each function of the recommender system. As previously discussed, the blockchain feature selection process involves dividing features into 3 categories: compulsory, mandatory, and possible. Compulsory features are those that must be present in the blockchain platform for it to be considered. These features are typically critical to the platform’s functionality. Mandatory features, on the other hand, are those that are essential for a specific use case or application. They are not necessarily required for the platform to function, but they are necessary for the platform to be suitable for a particular purpose. Finally, possible features are those that provide additional functionality or value to the platform. They are not necessary for the platform to function, but they can enhance its performance or provide additional benefits.

**Decision map recommender system functions and their definitions.**

**Function and definition**

- Create the data set: blockchain platform and blockchain feature names are initially entered. Subsequently, the platforms are associated with their corresponding features.
- Select features and their categories and set weights: specify the category of the blockchain feature by selecting 1 of the 3 options, namely, compulsory, mandatory, or possible. Then, assign the selected feature to the designated category. In addition, assign weights to the mandatory and possible features.
- Obtain top platforms: retrieve blockchain platforms with *compulsory features* and count the number of mandatory and possible features found for each platform. Calculate a score for each platform based on its features and weights and add it to an array. Sort the array based on the calculated score to display the top-performing blockchain platforms.

#### Creating the Data Set

**Figure 7:**
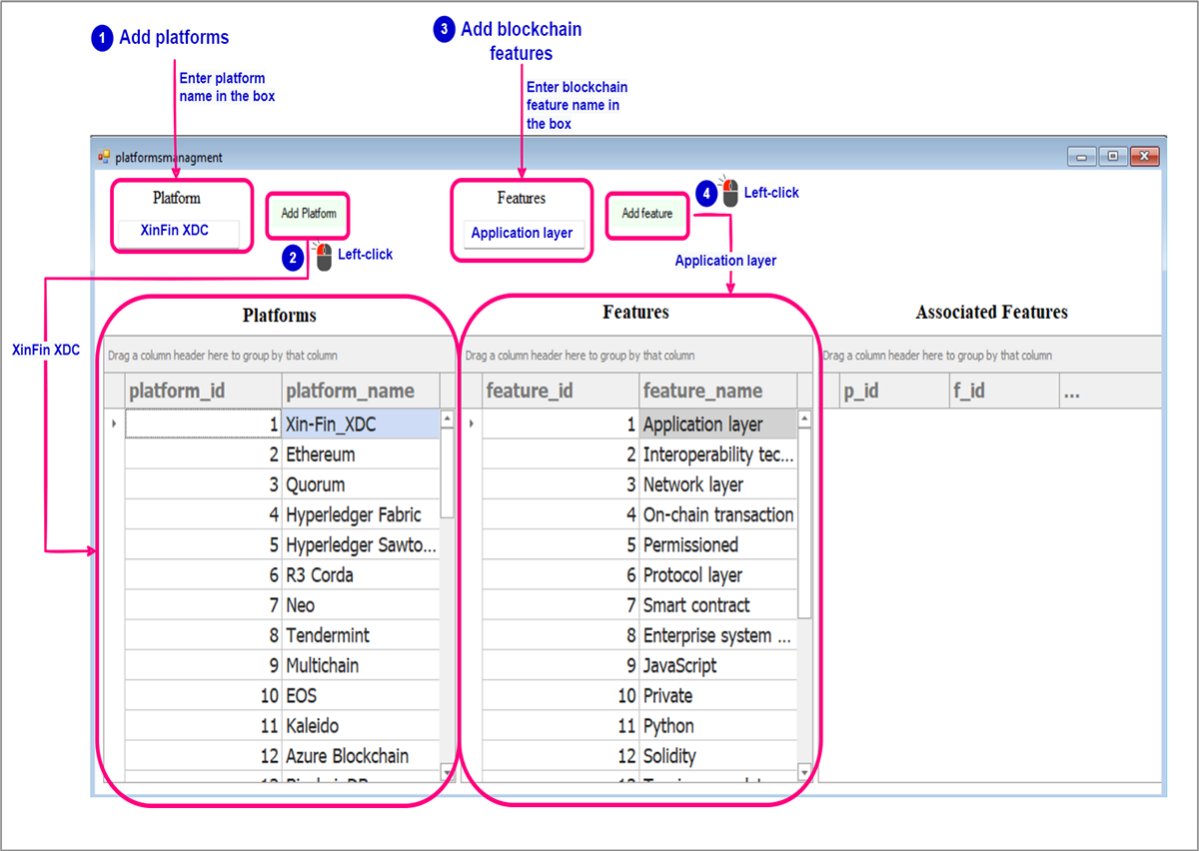
Data set creation—step 1: adding platforms and blockchain features that will be used to create a data set.

**Figure 8:**
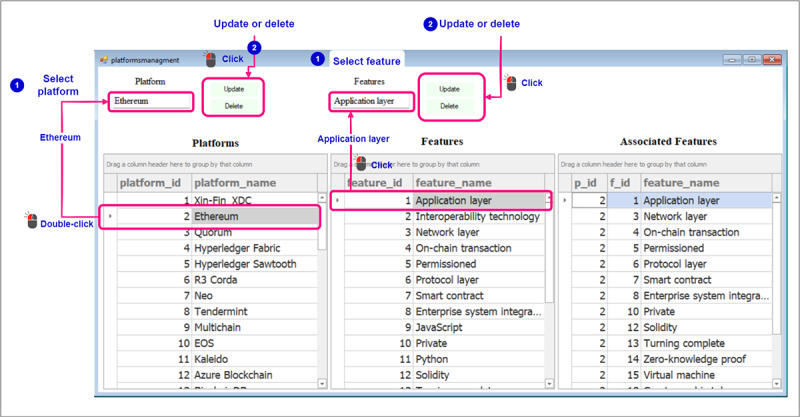
Data set creation—modifying and removing platforms and features from the data set.

**Figure 9:**
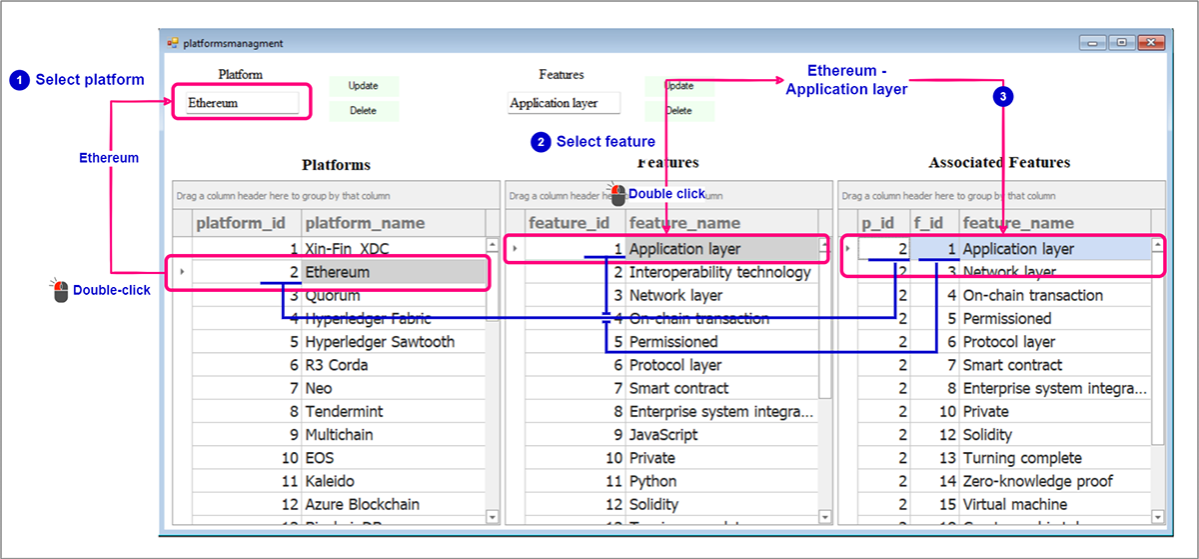
Data set creation—linking specific features with corresponding blockchain platforms.

This section illustrates the data set creation process, including user interactions with the recommender system. The blue annotations in the figures represent instructions. The pink annotations indicate the textboxes for input, buttons for actions, and grid controls for displaying the added platforms and features. In this step, we focused on adding the necessary platforms and blockchain features to build a comprehensive data set. Figure 7 shows the user’s process of entering the platform name and selecting *Add Platform* to populate a table showing the added platforms. The same procedure applies to adding blockchain features, resulting in a comprehensive table showcasing both platforms and features. After that, users are provided with the capability to edit platform names or delete them, as well as modify the names or choose to delete blockchain features. As shown in Figure 8, by double-clicking on a platform and single-clicking on a feature, users can select and make changes to the respective names according to their preferences. After that, users should establish the association between each blockchain platform and its corresponding blockchain features. They can begin by selecting a platform by double-clicking on the row corresponding to the platform name and subsequently choosing the blockchain features that apply to that particular platform, which is done by double-clicking on the rows that correspond to the blockchain features that should be mapped to the selected blockchain platform (Figure 9). Subsequently, a table will be populated with the IDs of the selected blockchain platform, the chosen blockchain feature, and the name of the blockchain feature.

#### Select Features and Their Categories and Set Weights

**Figure 10:**
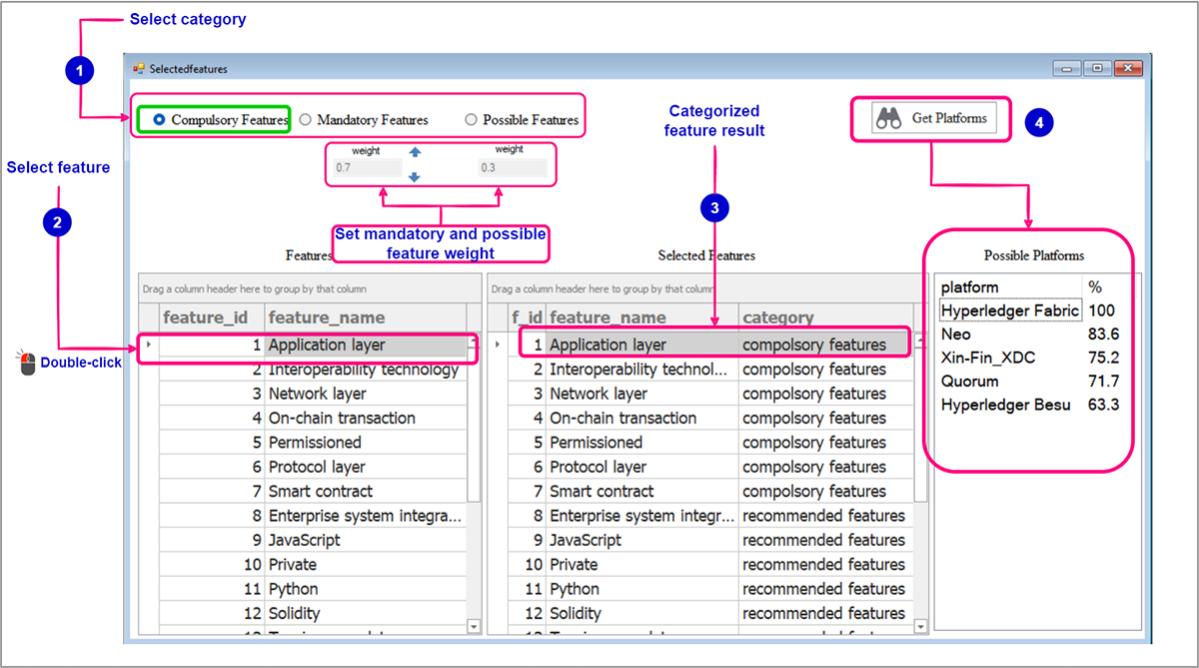
Allowing for specification of preferred blockchain features followed by categorization and weight assignment.

Figure 10 shows a screenshot illustrating user interaction during the process of selecting features, assigning them to their respective categories, and assigning weights to those categories. In the initial step, users will choose a category, followed by selecting the desired feature to be assigned to that category. This selection process involves double-clicking on the feature name in the table. Subsequently, a table will display the categorized features, providing a clear overview of the features that have been assigned to their respective categories. Once the categorization of features is complete, users can proceed to set the weights for the mandatory and possible feature categories. Afterward, by clicking on the *Get Platforms* button, users can view the resulting platforms based on the assigned weights and feature categorization.

#### Obtaining the Top Platforms

**Figure 11:**
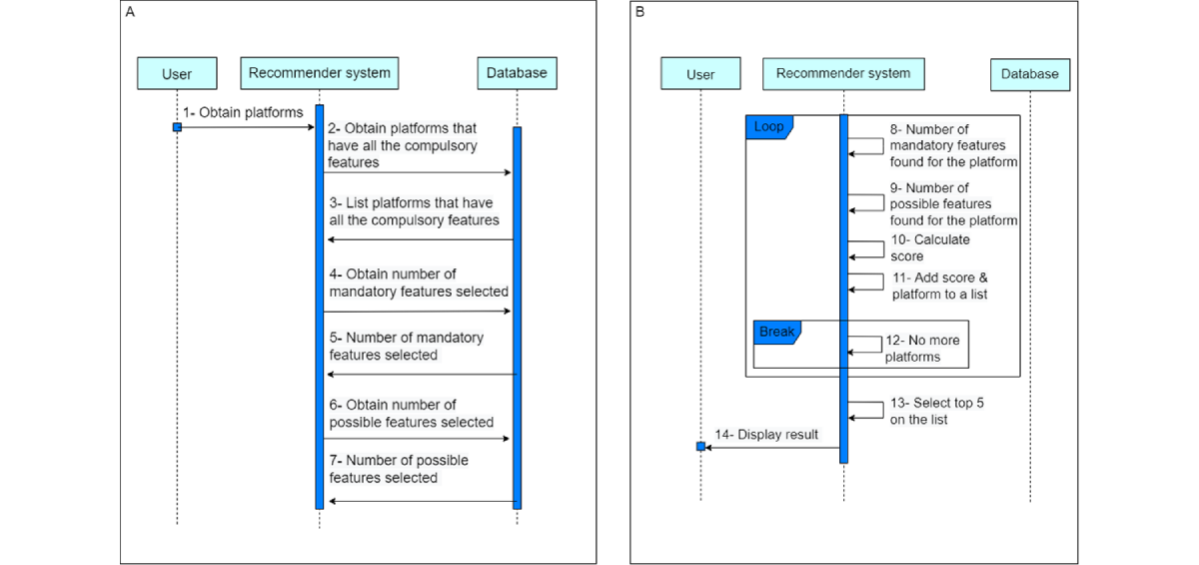
Diagram to obtain the fitness of the possible blockchain platforms for the use case.

Figure 11A and B show the sequence diagram to obtain the suitability percentage of each possible blockchain platform. The initial step involves creating a list of blockchain platforms that meet the requirements of the compulsory features. Once that is done, we determine the total number of mandatory and possible features that have been chosen (Figure 11A).

After that, we iterate through the list, and for each platform, we calculate the number of mandatory and possible features (Figure 11B). Finally, using equation 1, we calculate the suitability percentage of each platform (ρ). The first part of the formula calculates the contribution of the mandatory features to the suitability percentage. It takes the number of mandatory features found for the platform (*M_found_*), multiplies it by 100 to convert it to a percentage, and then divides it by the number of mandatory features selected (*M_total_*) multiplied by the weight assigned to mandatory features (ω*_M_*), which is 0.7.

The second part of the formula calculates the contribution of the possible features to the suitability percentage. It takes the number of possible features found for the platform (*P_found_*), multiplies it by 100 to convert it to a percentage, and then divides it by the number of possible features selected (*P_total_*) multiplied by the weight assigned to possible features (ω*_P_*), which is 0.3.

By combining these 2 contributions, the suitability percentage provides an overall assessment of how well a blockchain platform meets the selected features, with a higher percentage indicating a better match.



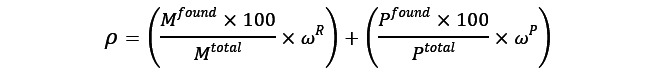



**(1)**

**Figure 12:**
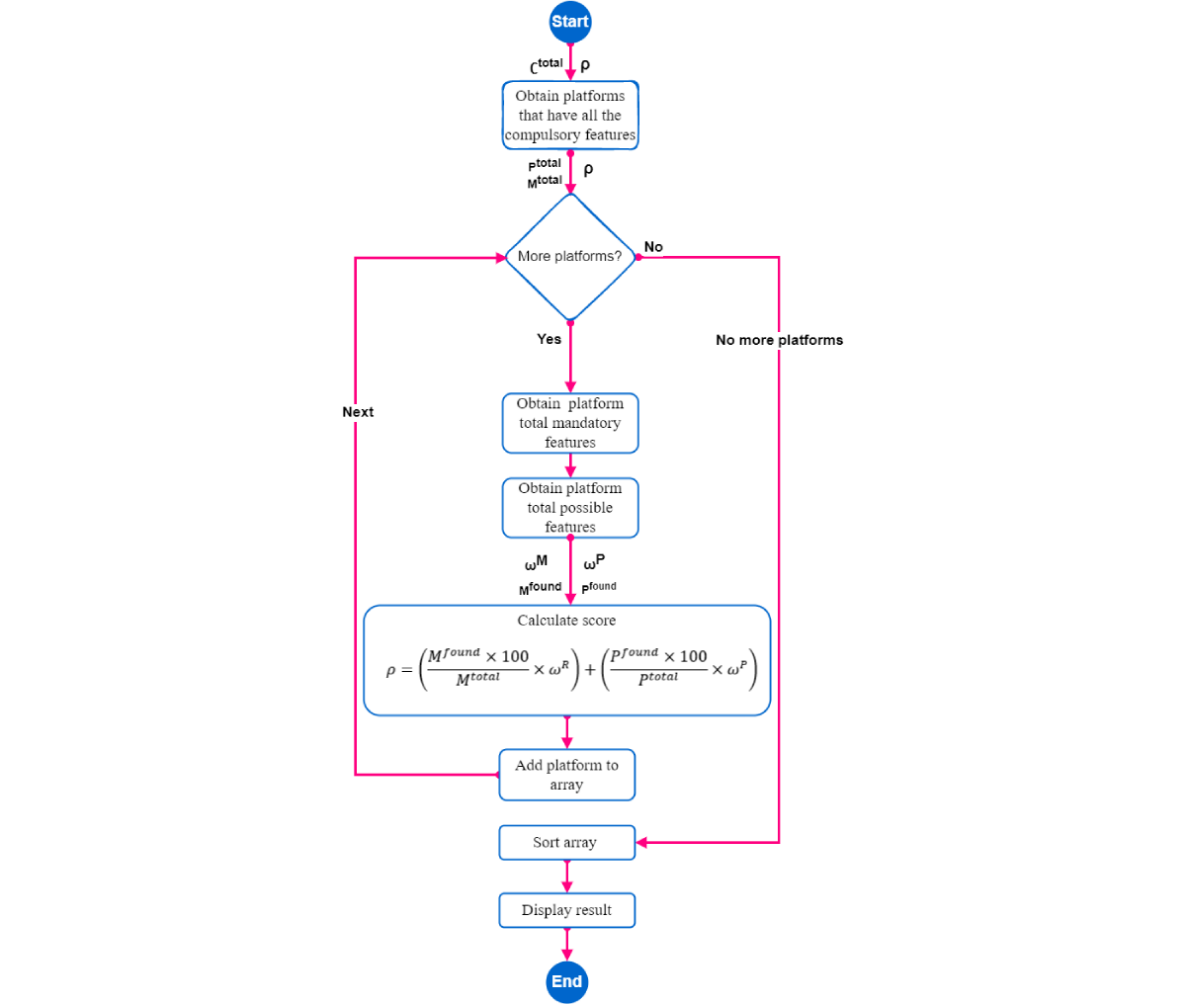
Flowchart to obtain the fitness of the possible blockchain platforms for the use case.

Once we have calculated the suitability percentage (ρ) for each platform, we sort the list of platforms in descending order based on their scores. Figure 12 shows the flowchart of the recommender system’s algorithm, which consists of the different functions involved along with their corresponding input and output parameters.

The platform with the highest score will be at the top of the list, whereas the one with the lowest score will be at the bottom. Finally, we display the top 5 platforms in the list, which are the ones that have the highest scores and, therefore, are the most suitable based on the selected features.

### Smart Contracts for Health Care Insurance Fraud Detection

**Figure 13:**
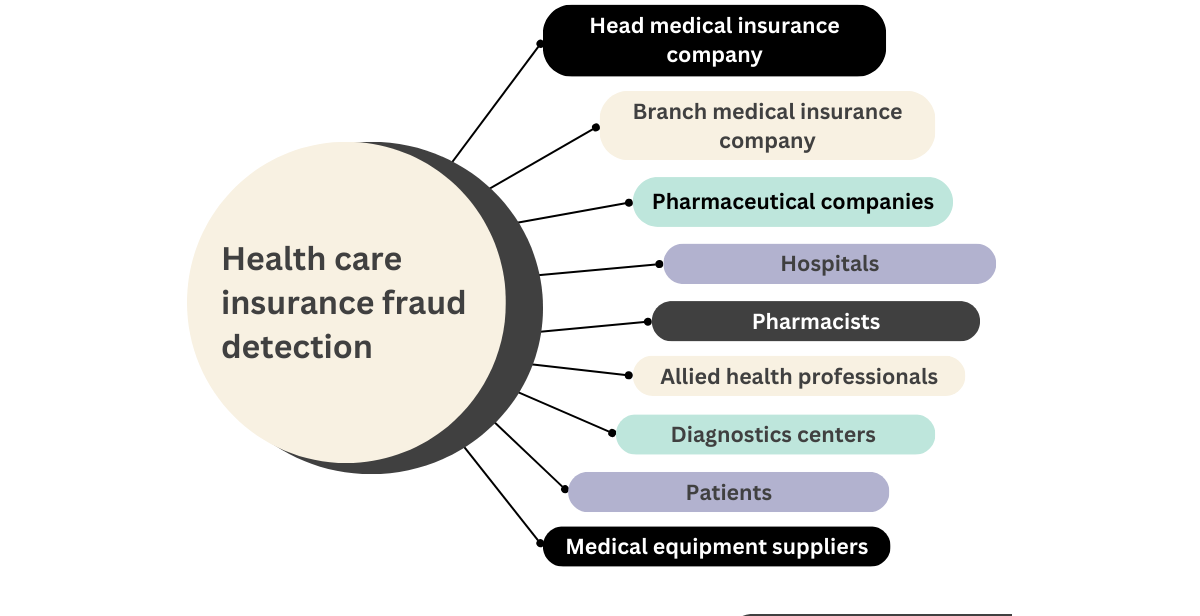
Participants in the health care insurance fraud detection network.

Ismail and Zeadally [22] identified the fraud scenarios used for detecting health care insurance fraud, as shown in Figure 1. The network for detecting health care insurance fraud is made up of 9 participants, as illustrated in Figure 13.

### Algorithms for Fraud Scenarios

#### Overview

For certain fraud scenarios, we need to discover a detectable pattern, whereas for others, data from off-chain sources may be required. The required data for processing claims consist of detailed records of patient visits, including the dates in which they occurred, the departments involved, the services rendered, and patients’ information. Consequently, they are on-chain. However, documentation of billed services, detailed service invoices, and pharmacy records are off-chain in the database.

#### 3 Referral Fraud Scenarios

**Figure 14:**
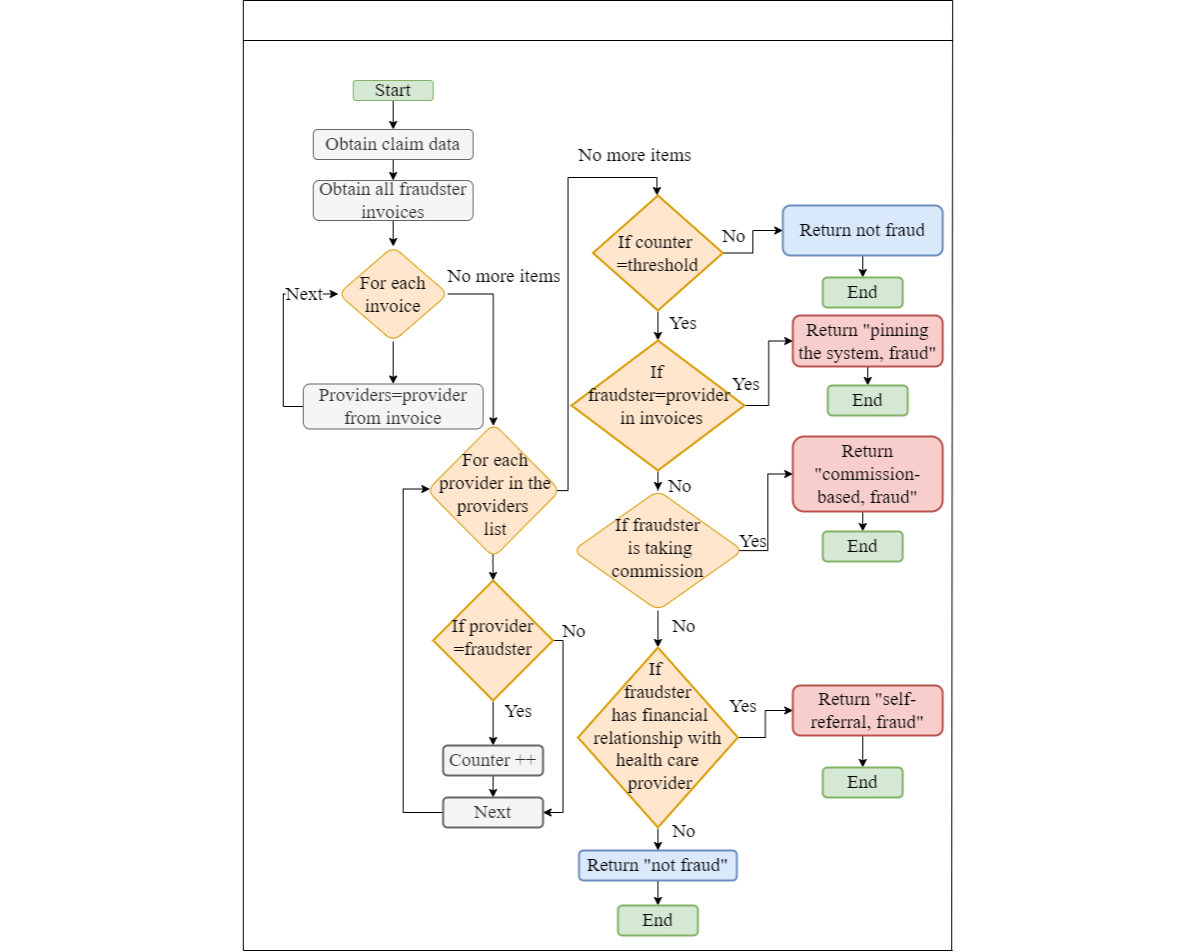
The 3 fraud scenarios related to referrals.

As shown in Figure 14, we use an algorithm to recognize 3 fraud scenarios that have the same pattern—the referral. We then check for the first scenario, in which the fraudster refers patients within the same health care organization. If this is confirmed, the fraud type is *pinning the system*. If not, we investigate whether a financial relationship exists between the fraudster and the other organizations. If such a relationship is detected, it is self-referral fraud. If no financial relationship is found, we investigate whether the fraudster received a commission from the organization; if so, it is a commission-based fraud.

#### Commission-Based Fraud

**Figure 15:**
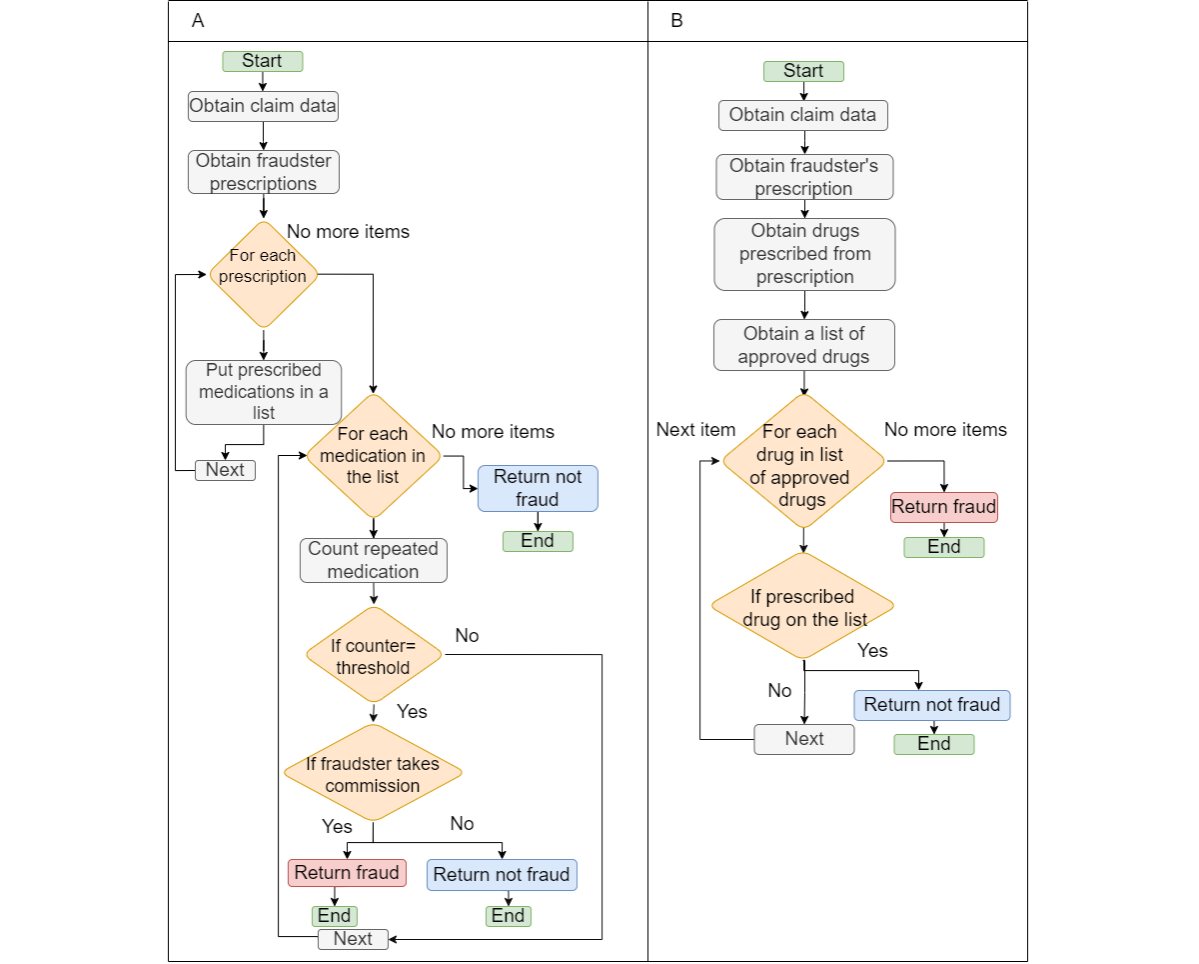
(A) Providing specific brands of medicines to receive a commission from the pharmaceutical company. (B) A pharmaceutical company provides incentives to physicians to promote unapproved or off-label drugs.

In this fraud scenario (Figure 15A), we obtain all the medication that the possible fraudster has prescribed. We then check whether a specific medication is prescribed more frequently and determine whether the fraudster is receiving a commission. If that is the case, it is a fraud. In Figure 15B, we should obtain a list from the minister of health containing the approved and labeled drugs and then compare it to the ones prescribed by the fraudster; if we find a drug that does not exist on the list from the minister of health, there is a fraud.

#### Managed Care and Waiving Copayment Fraud

**Figure 16:**
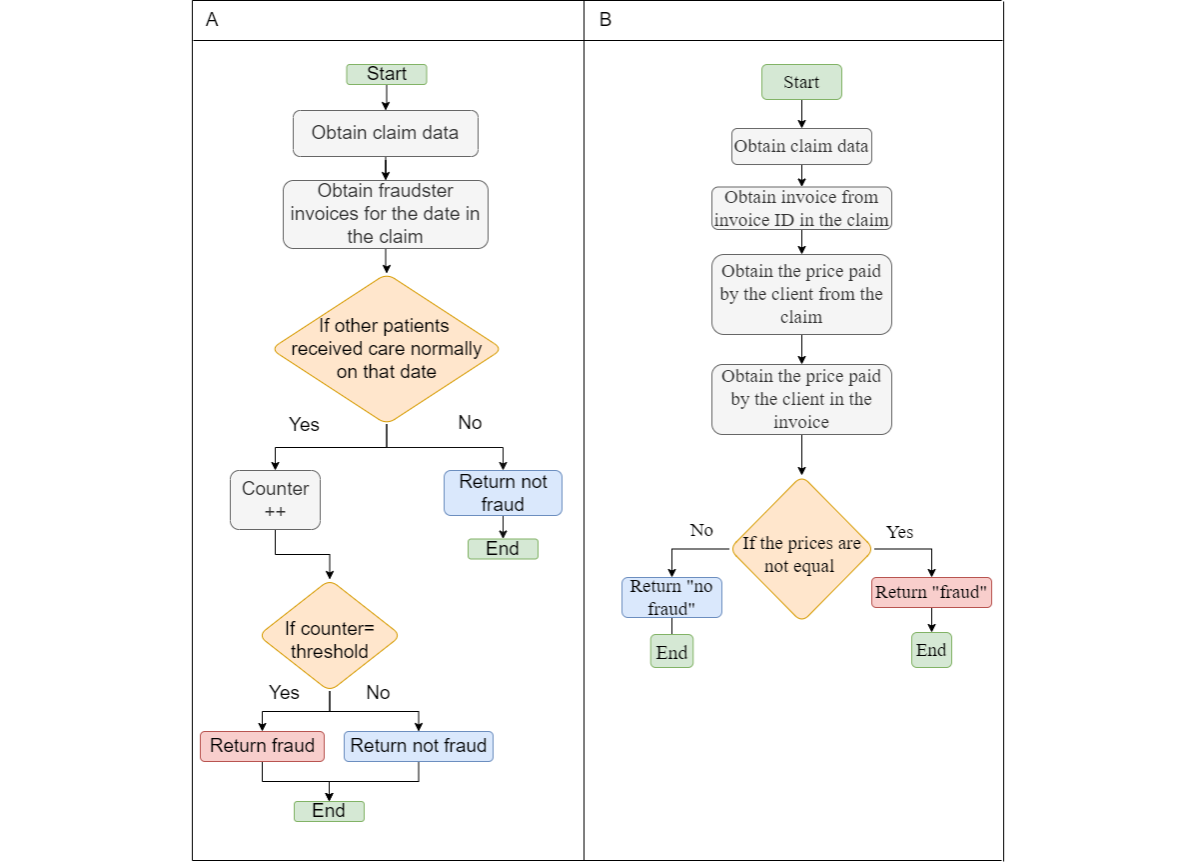
(A) Managed care and (B) waiving copayment fraud.

In Figure 16A, we investigate whether other patients on that date received the same service that the patient requested, and if the number of patients reaches a certain threshold, we are able to demonstrate that the managed care scenario is occurring.

The code for detecting waiving copayment fraud in Figure 16B involves comparing the price listed in the claim with the price mentioned in the corresponding invoice. If there is a mismatch between the 2 prices, it indicates a potential instance of waiving copayment fraud. The code performs a comparison operation to check whether the claim price and the invoice price are equal. If they are not, it raises an alert or triggers further actions to investigate the possibility of fraud.

#### Billing Manipulation Fraud

**Figure 17:**
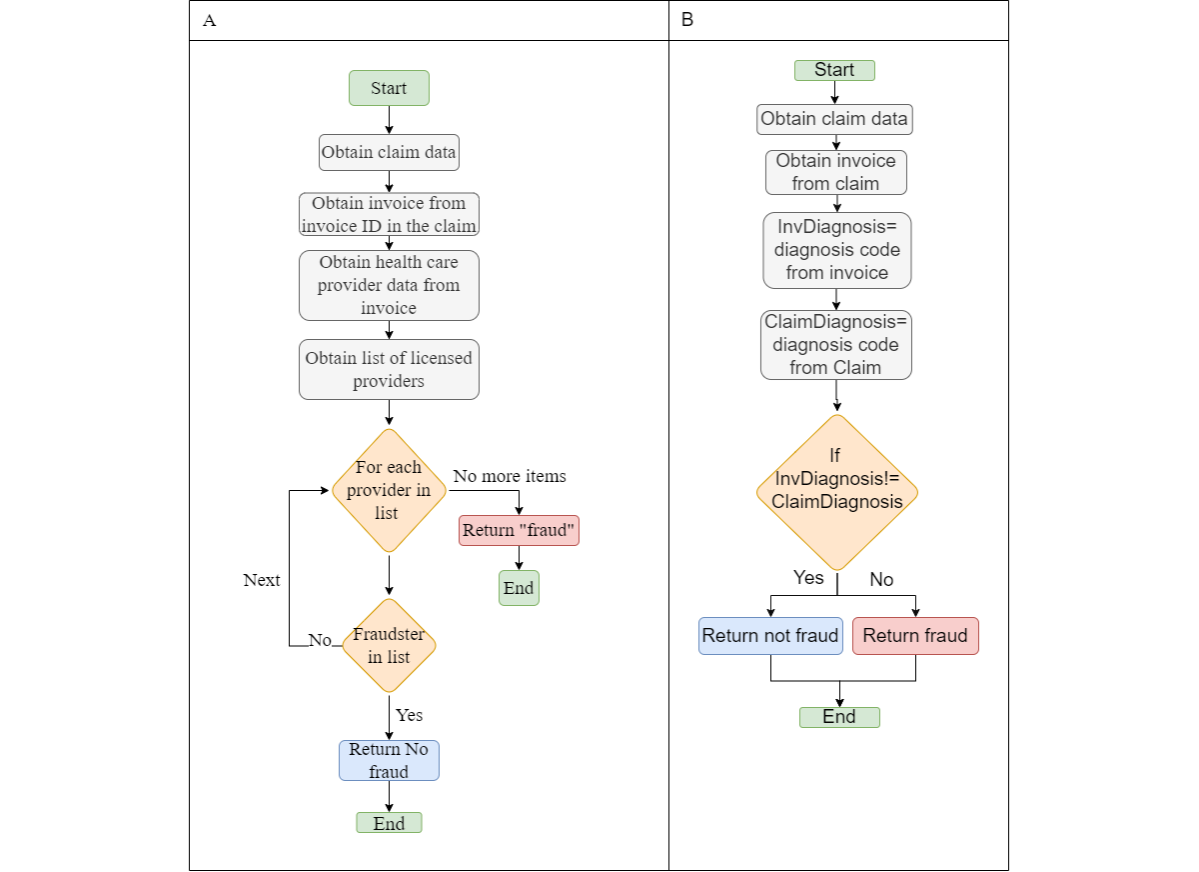
(A) Billing patients for care provided by an unlicensed care provider. (B) Manipulation of diagnosis in the claims without the knowledge of patients.

In Figure 17A, we obtain a list of licensed health care providers to determine whether the suspected fraudster is listed. If not, there is a case of fraud. In Figure 17B, we compare the diagnosis code on the claim to the one on the patient files; if they do not match, we will assume fraud.

**Figure 18:**
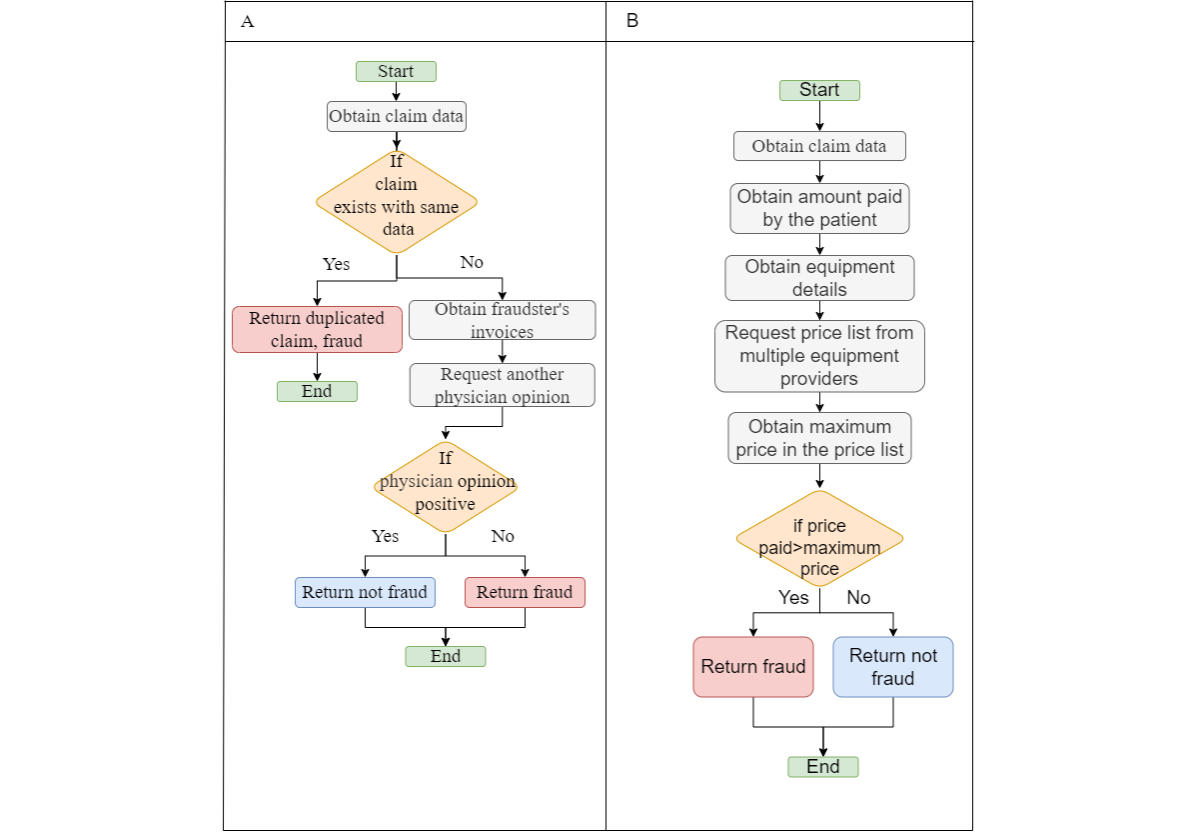
(A) Providing unwanted care to patients, increasing service hours in the bill, duplicating claims, phantom billing, or replacing codes of diseases with ones with higher prices. (B) Billing manipulation in equipment prices.

In this fraud scenario (Figure 18A), we may require the opinion of another physician, so after determining whether the claim has been duplicated, we gather all the necessary data to be reviewed by another physician, and based on the physician’s response, we determine whether the claim is fraudulent. In Figure 18B, we obtain a price list from other equipment suppliers and then compare it to the price paid by the patient; if it is higher than the price on the price list, the patient paid more than necessary for the equipment, and hence, it is a fraud.

#### Physician Shopping Fraud

**Figure 19:**
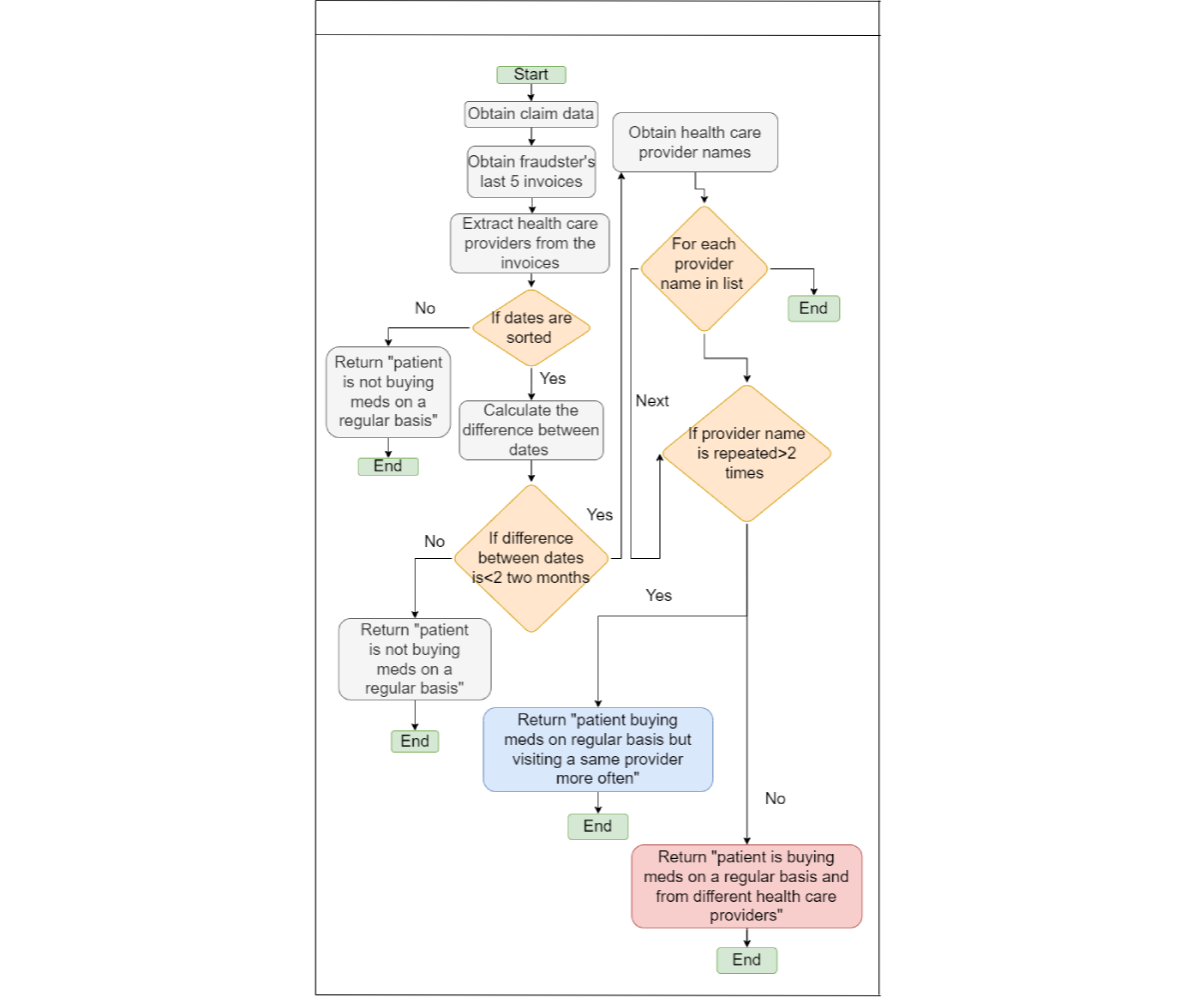
Physician shopping fraud.

Figure 19 illustrates physician shopping fraud, in which an addicted individual visits multiple health care providers to obtain unprescribed drugs. To detect this fraud, we must examine 5 invoices. We check whether the patient visits the provider regularly based on the dates from the invoices and whether the visits are not to the same provider. If this is confirmed, it is a case of fraud.

## Discussion

### Experiments

We created algorithms for smart contracts that address the fraudulent situations mentioned in the study by Ismail and Zeadally [22]. We used the 2 platforms that were selected as the top 2 options according to our decision-making recommender system. Each transaction contains a single claim record and requires supporting files such as invoices for verification. We assessed the performance of the platforms based on several metrics, including throughput, latency, CPU use, and memory use. Transaction throughput reflects the blockchain network’s efficiency in terms of the number of transactions processed per second. A transaction is considered successful once it has been included in a block and committed to the ledger. Transaction latency measures the time it takes to send a transaction request and receive a transaction response, indicating the network’s responsiveness. CPU and memory use are essential for determining infrastructure requirements and maintaining reliable performance under varying loads [31]. It is essential to ensure that a platform maintains high throughput, low latency, and minimal CPU and memory use [32]. This means that quality of service is maintained, and consequently, fraud is detected more quickly; costs are reduced; and, in some cases, patient lives are saved [33]. To evaluate the platforms’ effectiveness, we established 2 testing scenarios. The first scenario involved peers sending a consistent number of transactions over a period ranging from 30 to 120 seconds. In the second scenario, we progressively increased the number of transactions transmitted over the network from 1000 to 10,000.

### Experimental Environment

Our experimental setup involved using Ubuntu Windows Subsystem for Linux 2 (version 20.04; Canonical Ltd) on Windows 11 operating systems (Microsoft Corp). In the case of Fabric, we used Docker (Docker, Inc) to run the platform, and all peers were connected to a single channel representing an insurance company. The Fabric version we used was 2.2, with 4 organizations and 1 orderer. A batch size of 500 was set for processing transactions. We used Golang as the programming language for developing smart contracts.

For the Neo private blockchain, we used the N3 Neo Visual DevTracker extension on Visual Studio Code (Microsoft Corp). The programming language recommended in the Neo documentation, which is C#, was used. Similar to Fabric, a batch size of 500 was used. Both platforms used LevelDB as the key-value data storage.

In terms of hardware, we used a system with 16 GB of RAM and an 11th-generation Intel Core i7 processor running at 2.80 GHz. To conduct the benchmarking, we used Hyperledger Caliper [34] for Fabric and Neo-bench [35] for Neo.

### Security and Privacy Concerns

Health care data comprise sensitive patient information, making their security and privacy crucial. These data are typically stored in local or cloud databases. However, in such architecture, data face several issues regarding access and are subject to be deleted or modified and to cybersecurity attacks [14].

Using a blockchain-based approach addresses these concerns. Blockchain stores data in an immutable ledger where data can only be added after reaching a consensus and cannot be altered or modified. Changing a block or transaction impacts all subsequent blocks, and revalidating all subsequent blocks requires enormous computational power, making it nearly impossible for a malicious node. Furthermore, access control rights in blockchain can be defined in a smart contract, ensuring trustless and secure data access for network participants. Textbox 3 provides a concise overview of the challenges encountered in local on-premise or cloud database systems and demonstrates how blockchain technology addresses these issues.

**Comparison of issues in local on-premise or cloud databases versus blockchain for managing medical data.**

**Local on-premise or cloud database**

- Traditional record-keeping methods may not provide reliable auditing.
- Locally stored medical data might be unavailable in critical real-time situations.
- Local on-premise or cloud databases often lack proper authentication, leading to potential misuse of medical information.
- Local on-premise or cloud databases typically have less secure data access control.
- In a local on-premise or cloud database, unauthorized users can impersonate legal users to access sensitive medical data.
- Medical data in local databases can be easily altered or deleted.
- Without robust security, users could deny accessing or modifying data in local on-premise or cloud database systems.

**Blockchain**

- Blockchain’s replicated, time-stamped ledger facilitates efficient and trusted auditing.
- Data replication ensures real-time availability from the local copy of the ledger.
- Blockchain’s encryption and digital signature techniques ensure user authenticity for accessing and uploading medical information.
- Access control rights can be defined in smart contracts, ensuring secure and trustless data access for network participants.
- The private blockchain network restricts data access to authorized participants based on access control rights.
- Medical data are stored as transactions in blocks linked cryptographically to ensure immutability.
- Each operation’s authenticity is recorded in an immutable ledger, preventing repudiation.

### Results Analysis

Regarding the performance of Neo and Fabric in terms of throughput during the first test scenario, Fabric had 789 transaction per second (TPS), 445 TPS, 409 TPS, and 329 TPS, and Neo had 438 TPS, 629 TPS, 304 TPS, and 329 TPS during 30-second interval, 60-second interval, 90-second interval, and 120-second interval respectively. It is interesting to note that Neo initially experienced an increase in throughput, reaching a peak of 629 transactions per second (TPS) during the 60-second interval. However, it subsequently declines to 304 TPS and remains at that level for the remainder of the test. Similarly, Fabric follows a comparable pattern, starting with a high throughput of approximately 800 TPS and gradually decreasing to around approximately 450 TPS by the end of the test. This indicates that both platforms exhibited fluctuations in their transaction processing speeds throughout the test.

Regarding latency, it is worth noting that Neo consistently took approximately 14 to 24 seconds to confirm a transaction throughout the test duration.

The latency of Fabric in the first test scenario was 0.05 seconds, 0.12 seconds, 0.1 seconds, and 0.1 seconds during 30-second interval, 60-second interval, 90-second interval, and 120-second interval respectively. Here, we can observe that, as more transactions were submitted, the latency slightly increased. This implies that, as the workload on Fabric intensified with a higher number of transactions, the time taken to process and confirm each transaction also increased.

Regarding the throughput in the second test scenario, in which we increased the number of transactions sent over the network by 1000 up to 10,000 transactions. Fabric had 426 TPS, 470 TPS, 630 TPS, 577 TPS, 676 TPS, 707 TPS, 718 TPS, 732 TPS, 740 TPS, and 754 TPS. Neo had 373 TPS, 427 TPS, 489 TPS, 422 TPS, 534 TPS, 543 TPS, 434 TPS, 424 TPS, 409 TPS, and 474 TPS. Fabric outperformed Neo once again. Fabric demonstrated an upward trend in throughput as the number of transactions increased, whereas Neo exhibited more fluctuations in its performance.

Regarding the latency in the second test scenario, Fabric had 0.03 seconds for 1000 TX sent, 0.04 for 2000 TX, 3000 TX, 4000 TX and 5000 TX, 0.03 seconds for 6000 TX, 7000 TX, 8000 TX and 9000 TX, and 0.04 for 10,000 TX. The latency for Fabric remained relatively stable, with a slight increase observed. The latency values consistently ranged between 0.03 and 0.04 seconds throughout the test. This indicates that Fabric can maintain low and consistent latency even as the number of transactions increases.

Delays in fraud detection can slow the identification of fraudulent activities, causing financial losses and putting patients’ health at risk. High latency impedes the prompt discovery of fraud, giving wrongdoers the chance to persist in their schemes unchecked, possibly resulting in more harm and greater financial damage. Thus, reducing latency is essential to improve the efficiency and precision of fraud detection, ultimately protecting health care resources, patients’ health, and the credibility of health care services [36]. Throughput and latency can be significantly impacted by large transaction sizes, which are driven by extensive file requirements and block size. In private blockchain networks, the computational complexity and energy consumption of encryption and decryption operations add to this burden. Furthermore, replicating the ledger across all nodes increases computational and network overhead, resulting in high energy consumption, low transaction throughput, and limited scalability. As the number of nodes increases, so does the volume of data transferred, leading to longer processing times. In addition, the choice of consensus mechanism affects scalability; for instance, proof of work is particularly known for its high energy consumption, further exacerbating these challenges.

The CPU and memory use comparison between Fabric and Neo reveals that Fabric used fewer resources than Neo. Fabric utilized 191 MB of memory and 47% of the CPU, while Neo used 515 MB of memory and 39% of the CPU. Throughout both conducted tests, both platforms achieved a 100% success rate, indicating their reliability for securely sharing sensitive health care insurance data. Fabric consistently outperformed Neo in both tests, showcasing its superiority in creating health care insurance fraud detection systems. The lower resource consumption by Fabric suggests that it offers more efficient resource use, making it an optimal choice for health care insurance fraud detection applications.

### Limitations

Validating the system using real-world health care data presents several challenges and ethical considerations. Obtaining access to real-world health care data can be difficult due to stringent regulations and privacy concerns. Ensuring that the data are accurate, complete, and representative of the broader population can be challenging as inconsistent or incomplete data can affect the validity of the results. Integrating diverse data sources and formats into a cohesive system requires significant technical expertise and resources. From an ethical standpoint, protecting patient confidentiality is paramount, necessitating robust measures to ensure that data are anonymized and secure. In addition, there is a risk of introducing bias if the data are not representative of the entire population, potentially leading to skewed results and harmful recommendations.

In addition to the technical and ethical considerations, the successful implementation of this system requires the cooperation and acceptance of various participants in the health care system [12]. Key stakeholders such as hospitals, clinics, insurance companies, and health care providers must be willing to contribute their data and support the integration of a blockchain-based solution. Each participant has unique requirements for data security, privacy, and interoperability that must be addressed to ensure their cooperation. Furthermore, patient data must be governed and secured to ensure privacy and controlled access. Only authorized personnel should be able to access sensitive information, and all data handling procedures should comply with relevant regulations [37].

### Conclusions and Future Work

Health care insurance fraud detection is crucial for the health care industry. This is due to the high costs incurred from health care insurance fraud. In addition, some frauds pose risks to patient health. In this study, we designed and implemented smart contracts to detect health care insurance fraud. This is based on our proposed taxonomy of fraud scenarios. Furthermore, we used a blockchain platform that is specifically suited for health care insurance fraud detection. To improve the selection of a suitable platform, we designed and implemented a decision map–based recommender system, which automates and streamlines the platform selection process. To feed the recommender system with suitable candidates, we proposed a taxonomy of 102 blockchain development platforms. Through these efforts, we aimed to improve the efficiency and accuracy of health care insurance fraud detection by leveraging the capabilities of blockchain technology. The recommender system revealed Fabric and Neo as the top 2 platform candidates for the development of health care insurance fraud detection solutions, with Fabric having the highest rank. Our experimental numerical evaluation of the 2 selected platforms showed that Fabric outperformed Neo, demonstrating a more suitable network structure and features than Neo. Furthermore, based on our experiments, Fabric offers greater configurability, enabling further performance improvements. Machine and deep learning algorithms are alternative promising approaches for detecting patterns of fraud in large-scale environments such as health care insurance. However, these algorithms suffer from security and privacy issues and are prone to bias. In future work, we aim to integrate machine learning techniques for health care insurance fraud detection into blockchain. This integration would provide trust in the machine learning techniques, and aid their traceability and understanding, leading to privacy-preserving machine learning models. In particular, we will explore how blockchain can improve the privacy and security of machine learning models, investigate the most effective ways to integrate federated learning with blockchain to ensure that data remain decentralized and secure, and design smart contracts to automate and verify the training and deployment of machine learning models.

## Abbreviations

CPU: Central Processing Unit
TPS: transactions per second
